# State Marine Aquaculture Policy Dashboard improves transparency and accessibility for growing industry

**DOI:** 10.1371/journal.pone.0310602

**Published:** 2024-09-18

**Authors:** Elizabeth O. Ruff, Stephanie Showalter Otts, Hayley R. Lemoine, Rebecca R. Gentry, Sarah E. Lester

**Affiliations:** 1 Department of Geography, Florida State University, Tallahassee, Florida, United States of America; 2 National Sea Grant Law Center, University of Mississippi School of Law, Oxford, Mississippi, United States of America; 3 Department of Biological Science, Florida State University, Tallahassee, Florida, United States of America; Central Marine Fisheries Research Institute, INDIA

## Abstract

Marine aquaculture (mariculture), the farming of marine species, is currently a relatively small contributor to the United States’ seafood industry. There is tremendous potential for growth in this sector and increasing interest in advancing the industry through supportive federal and state policies as well as concerns about how to best manage potential negative impacts or unsustainable development. While some mariculture is conducted in land-based tanks as well as saltwater ponds, and there are pilot projects and ongoing applications to commence mariculture operations in federal waters, the majority of mariculture activities occur within state waters (typically, 3 nautical miles, or 5.5 kilometers, from shore) and are largely managed by state-level policies and regulations. The policy mechanisms by which each of the 23 coastal states manages their respective mariculture industries are quite varied, making it difficult to identify policy trends and assess which approaches may be enabling or impeding the development of the sector. As such, we present the State Marine Aquaculture Policy Dashboard: a publicly-available, living database collating state-level policy and legislative data related to the management of the mariculture industry. This centralized, accessible catalog of laws, policies, regulations, and initiatives is a valuable resource for understanding the current landscape of state mariculture policy frameworks in the U.S. and can create opportunities for policy transfers and collaboration across states as they seek to manage their industries.

## 1. Introduction

As a relatively nascent commercial industry in the United States, the state-level policies and legislation governing the farming of marine species (known as marine aquaculture or mariculture) in ocean spaces (as opposed to land-based tanks or saltwater ponds) are still evolving. With characteristics of both capture fisheries and livestock production, the mariculture industry is often guided by a broad spectrum of fisheries, agriculture, environmental, and food health and safety policies and regulations across multiple federal and state government agencies [1, 2]. Further, the process for authorizing mariculture activities and leasing ocean spaces for farm infrastructure can be lengthy and complicated, requiring multiple agency approvals and permits [3–5]. The decentralized and complex nature of these regulatory frameworks is often cited as a barrier to the development of the industry in the U.S. [1, 6]. While the industry is subject to twenty federal laws and some federal permits are necessary to commence operations, the majority of mariculture activities occur within state waters (typically, 3 nautical miles, or 5.5km, from shore) and, as such, are predominantly managed by state-level policies, regulations, and processes [7]. Even at the state level, navigating the dynamic mariculture policy landscape can be difficult for policymakers, industry regulators, seafood farmers, and researchers alike. Particularly in states where mariculture is governed by a patchwork of legislation and regulations enforced by different agencies, it can be difficult to develop streamlined and effective policy, which can impact the pace of industry growth and create inequities with regard to who can participate in and benefit from the industry [8, 9].

Increasing knowledge of and access to states’ mariculture policies improves transparency within and across the regulatory landscapes of each state, which can support effective industry management and reduce barriers to participating in the industry [3]. Here, we introduce the State Marine Aquaculture Policy Dashboard: a publicly-available, living database collating state-level policy and legislative data related to the management of the mariculture industry. The Dashboard was developed by a collaborative team of researchers at Florida State University and staff at the United States National Oceanic and Atmospheric Administration’s Sea Grant Law Center (including the present authors) and will continue to be maintained through this partnership.

In this paper, we employ ‘policy’ as a broad term for legislation, statutes, regulations, practices, management frameworks, and government agencies and programs that oversee and facilitate mariculture activities within each state. Previous efforts to collate state-level mariculture policy data in the U.S. have produced summary reports, published papers, and the consolidation of qualitative data into large, dense spreadsheets (e.g., NOAA, 2021 [10]). Lester et al. (2021) conducted a systematic overview of state-level mariculture policy, compiling information for 16 policy ‘attributes’ across the 23 coastal states, with a focus on attributes that could enable industry development [2]. However, given how quickly the management of the mariculture industry is evolving, the information provided by previous work can—and some of it has—become quickly outdated. Further, the Lester et al. (2021) review was primarily focused on enabling policy, leaving out information on some important attributes that may limit or restrict development [2]. We also note that these previous efforts lack visualizations and the ability to easily compare information across multiple states, making it difficult to explore patterns in the data and identify opportunities for knowledge transfers.

**Fig 1:**
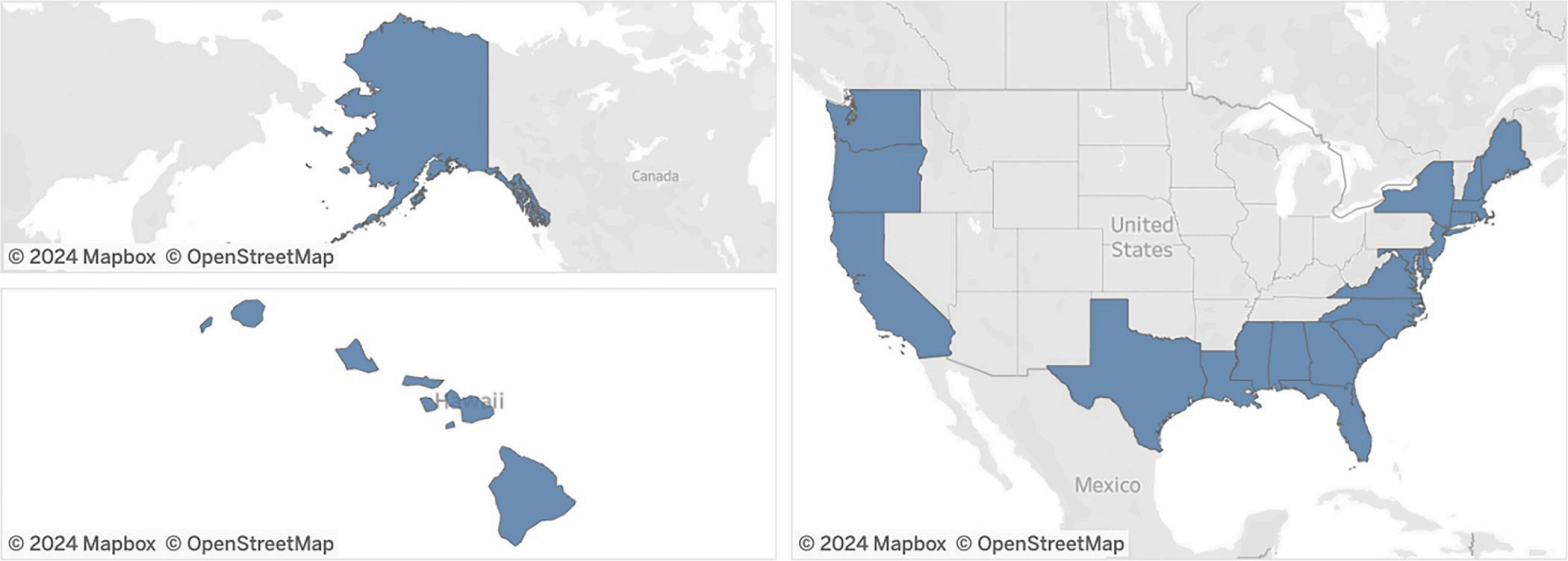
Coastal states (N = 23) included in the State Marine Aquaculture Policy Dashboard. This Dashboard is made available under the Open Database License: http://opendatacommons.org/licenses/odbl/1.0/. Any rights in individual contents of the database are licensed under the Database Contents License: http://opendatacommons.org/licenses/dbcl/1.0/.

The State Marine Aquaculture Policy Dashboard (accessible at https://bit.ly/nsglc-tableau) improves on these shortcomings by providing a living, interactive database that can be easily updated. The Dashboard collates state-level mariculture policy, legislative, and industry management information for the 23 coastal states (Fig 1). As of July 2024, the Dashboard includes data for 42 legislative, policy, and management attributes—including the 16 attributes from Lester et al. (2021) [2]—spanning industry regulations, agency authority, ocean leasing, farming operations, spatial management tools, biosecurity, sustainability, tribal authority, and capacity building. This expanded dataset provides a more comprehensive review of state’s existing policy frameworks, enabling and otherwise.

Designed to support the varied needs and objectives of policymakers, regulators, and industry participants, the Dashboard offers multiple visualization tools and methods of interacting with the data. It is also a rich resource for researchers exploring policy dynamics within the industry, as each state provides a natural case study for assessing the effects of different policy approaches. By increasing the transparency of and accessibility to mariculture policies, practices, and resources, the Dashboard is intended to encourage greater collaboration both within and between states and agencies to develop and disseminate supportive frameworks for industry development and sustainability.

## 2. Materials and methods

### 2.1 Attribute selection

We selected attributes based on existing published research, mariculture policy, and management frameworks as well as conversations with mariculture producers, regulators, and extension agents. From this process, we identified 38 possible new attributes to include in the Dashboard. We further refined this list to 26 new attributes based on three criteria:

1. **Feasibility of uniform data collection**: We prioritized attributes for which we could collect cohesive data across states. Having comparable data for each attribute supports the use of the Dashboard as a tool for knowledge transfers and policy evolution.
2. **Practicality to the Dashboard’s target audience of policymakers, regulators, and industry participants**: Through our conversations with stakeholders and our own knowledge of mariculture activities, we identified key mechanisms that are most relevant to the regulation of the industry and operation of mariculture farms. These attributes are especially important to improving regulatory transparency.
3. **Potential interest to mariculture researchers**: The policy and management dynamics of mariculture in the United States are an increasingly popular and substantive field of research. In assessing existing mariculture research and policy, we identified key gaps (e.g., biosecurity regulations [per communications with state aquaculture managers]) as well as frequently highlighted topics (e.g. capacity building [1, 11, 12]), and we included those that also met the first criterion above in the Dashboard.

The Dashboard comprises these 26 new attributes as well as the 16 attributes from Lester et al. (2021) for a total of 42 attributes [2]. S1 Table presents all of the attributes included in the Dashboard and their descriptions.

### 2.2 Data collection and collation

We collected data from primary sources for all attributes and states. These primary sources included state statutes and regulations, state agency policies, state government websites, and other state government-supported resources. For most attributes, we recorded binary responses (yes or no) based on whether a given state currently has an existing law, regulation, policy, practice, or resource that matches the criteria outlined in S1 Table. The few exceptions to this process are the attributes for ‘agency lead: permitting’, ‘agency lead: leasing’, and ‘maximum lease term’, for which we recorded agency names and numeric values for the lease term. In addition to these binary, nominal, and numerical data, we provided additional detail(s), when relevant, regarding the way a given state addresses a specific attribute. For example, in addition to identifying whether or not a state requires a public consultation as part of its leasing process, we also include what form that public consultation takes (e.g., public hearing, public comment, public notice) and how many days the consultation period lasts. Additionally, the Dashboard also includes the original source of the data and, when appropriate, a link to that source so users can easily access the original information (e.g., law, regulatory code, government report, website, etc.) and more details.

### 2.3 Data validation

As highlighted above, the policy and management landscape for mariculture is complex and ever-evolving as the industry develops. As such, we reached out to mariculture managers from relevant government agencies in each state to review their respective data and provide corrections and feedback, when relevant. This outreach included a working session for state mariculture managers attending the (SMACN) Network Workshop in May 2023 as well as email correspondence with state managers that were unable to attend the workshop. To ensure consistent data coding across states, we evaluated any suggested revisions to ensure that they adhered to the policy attribute definitions and followed up with any clarifying questions. Verified revisions were updated in the Dashboard. To keep the data in the Dashboard current moving forward, we intend to reach out to this network of stakeholders (through both SMACN workshops and email) on an annual basis to review the existing data for their state and update us on any new developments.

### 2.4 Dashboard interface

While accurate and up-to-date data are important components of the Dashboard, we also prioritized the user interface to provide greater transparency and accessibility to the data than previous works provided [2, 10, 13]. The Dashboard interface, which uses Tableau Public, is specifically designed to address the needs and objectives of a broad range of user groups, including policymakers, regulators, industry participants, and researchers. We outline each of the Dashboard’s visualizations below, but we have also compiled a user guide posted on the National Sea Grant Law Center’s State Marine Aquaculture Policy Dashboard page (accessible at https://bit.ly/smap-dash-instructions) for a more detailed overview of how to use the Dashboard.

#### 2.4.1 Dynamic filters

**Fig 2:**
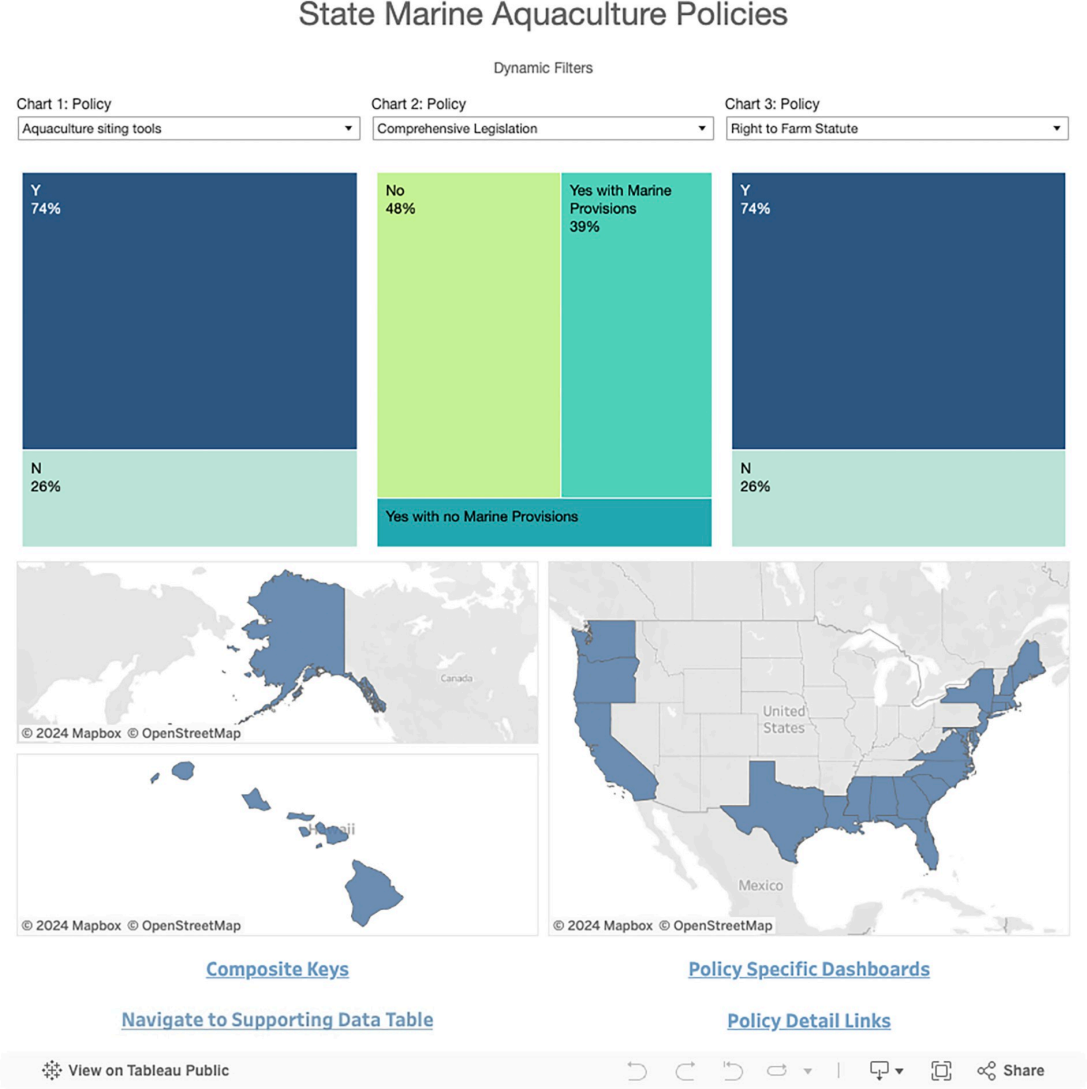
Homepage for the State Marine Aquaculture Policy Dashboard. This Dashboard is made available under the Open Database License: http://opendatacommons.org/licenses/odbl/1.0/. Any rights in individual contents of the database are licensed under the Database Contents License: http://opendatacommons.org/licenses/dbcl/1.0/.

**Fig 3:**
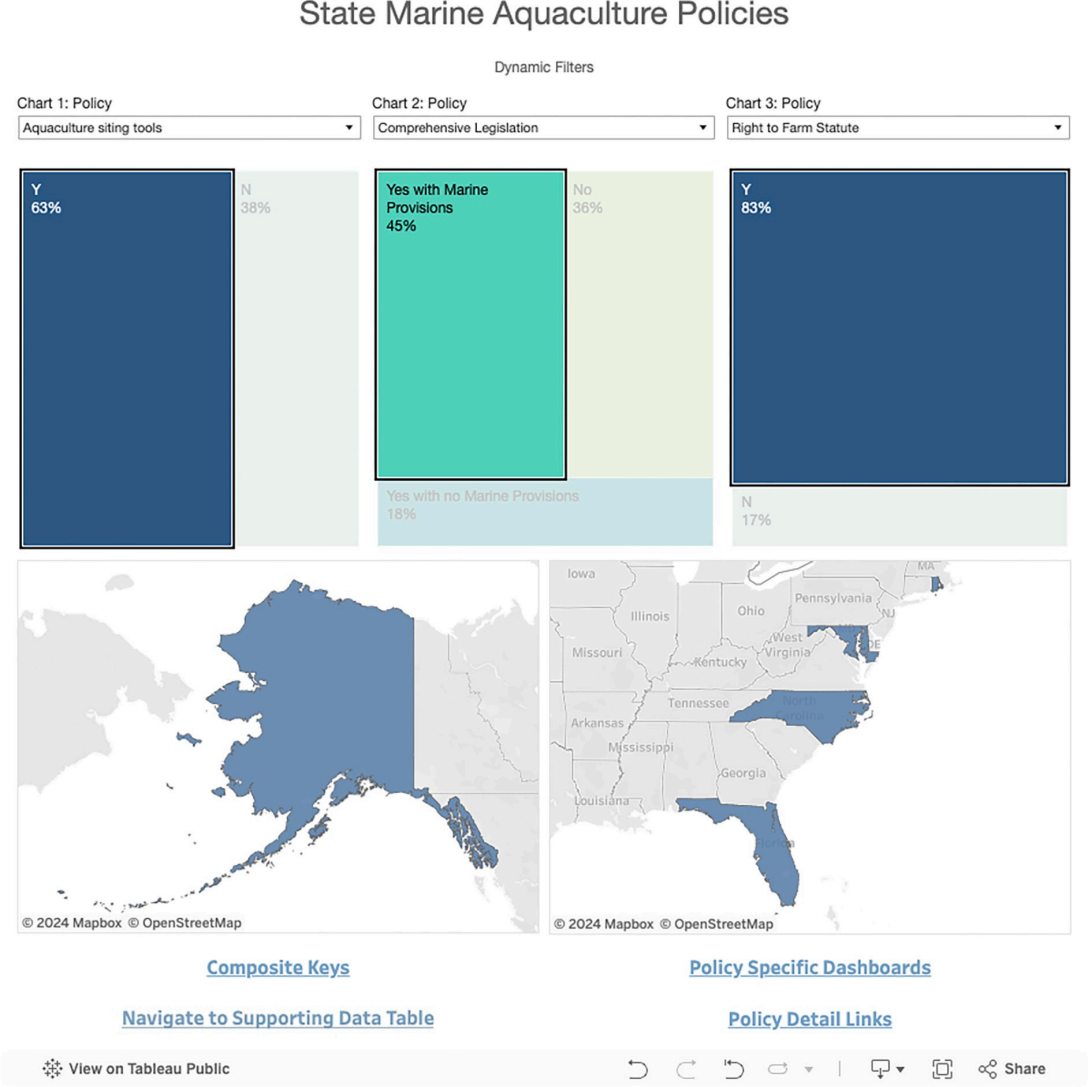
Example of filtering data by multiple policy attributes. The map shows the five states (Alaska, Florida, Maryland, North Carolina, and Connecticut) that have aquaculture siting tools, comprehensive aquaculture legislation with mariculture specific provisions, and right-to-farm statutes. This Dashboard is made available under the Open Database License: http://opendatacommons.org/licenses/odbl/1.0/. Any rights in individual contents of the database are licensed under the Database Contents License: http://opendatacommons.org/licenses/dbcl/1.0/.

The default dashboard ‘home’ view consists of dynamic filters for exploring the data in both chart and map form (Fig 2). Utilizing three dropdown menus, users can examine multiple policy attributes at once and explore connections among both attributes and states. Each dropdown menu has a corresponding chart directly below that displays the underlying data for that specific policy attribute. Users can further filter the data for a given policy attribute by clicking on a particular section (e.g., Y or N) within the corresponding chart for that attribute. This will apply that attribute as a filter both to the map below and the other two chart filters. For example, if users select the Y (‘yes’) section of a chart for aquaculture siting tools, only states with aquaculture siting tools will be reflected in the map and the percentages in the charts for the other two policy attributes. Users can filter even more by selecting policy attributes in the other two charts (Fig 3). This will allow users to narrow down to a specific state or subset of states that meet the chosen criteria. At the bottom of the Homepage, there are four links to additional data visualization options. *Navigate to Supporting Data Table* takes users to a viewable Google spreadsheet containing all of the underlying data for the dashboard, including the original source of the data. The other three visualization options are outlined in the following sections.

#### 2.4.2 Composite keys

**Fig 4:**
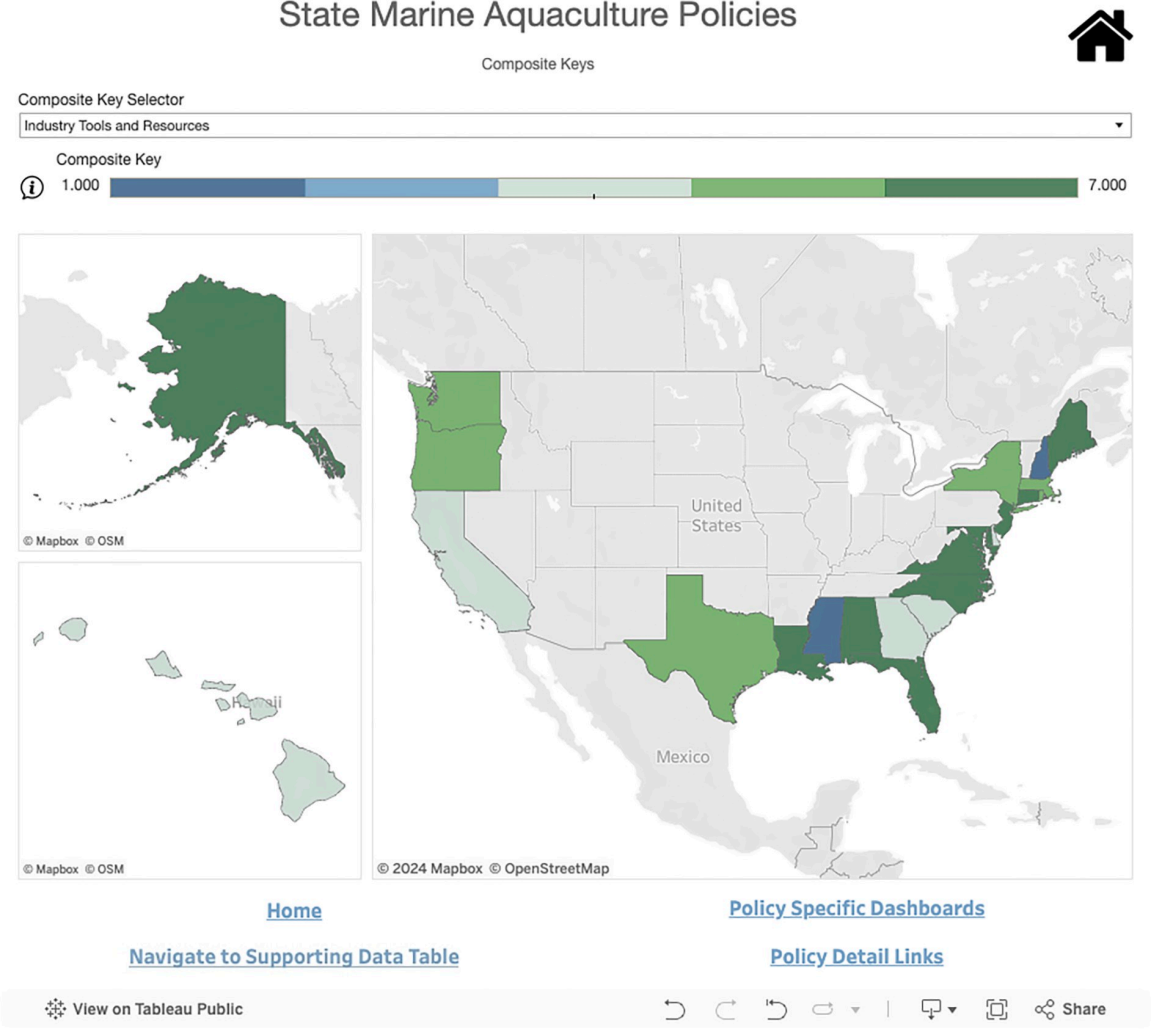
Composite key visualization for industry tools and resources. A dark blue color indicates a state has a lower composite score, and dark green indicates a higher score. This Dashboard is made available under the Open Database License: http://opendatacommons.org/licenses/odbl/1.0/. Any rights in individual contents of the database are licensed under the Database Contents License: http://opendatacommons.org/licenses/dbcl/1.0/.

The Composite Key visualization tool combines multiple policy attributes into a single index, or composite variable (Fig 4). This view allows users to explore the Dashboard’s data through pre-selected groups of related policies. The dashboard contains 3 Composite Keys for users to select from. The policy attributes included in each Composite Key are listed below:

- Environmental safeguards: native species requirements, species moratoriums, local population genetic requirements, reproductive requirements, aquaculture best management practices (BMPs), and bonding requirements
- Spatial management tools: zoning for marine aquaculture, aquaculture siting tools, and map of existing lease sites
- Industry tools and resources: regulatory guidance, government website for permitting, government website for leasing, centralized government website, aquaculture siting tools, map of existing lease sites, and training programs

Within a composite key, each attribute that is present for a given state contributes 1 point to the state’s score for that composite variable, with the total score depicted by the color ramp. A dark blue color indicates a lower composite score while a dark green color indicates a higher score. The more policy attributes a state has within a given composite key, the higher their composite score. Users can hover over the information icon to get more detailed information about the chosen indicator and composite attributes. Hovering over a specific state will show the individual policy attributes that contributed to its score for a given composite key.

#### 2.4.3 Policy specific dashboards

**Fig 5:**
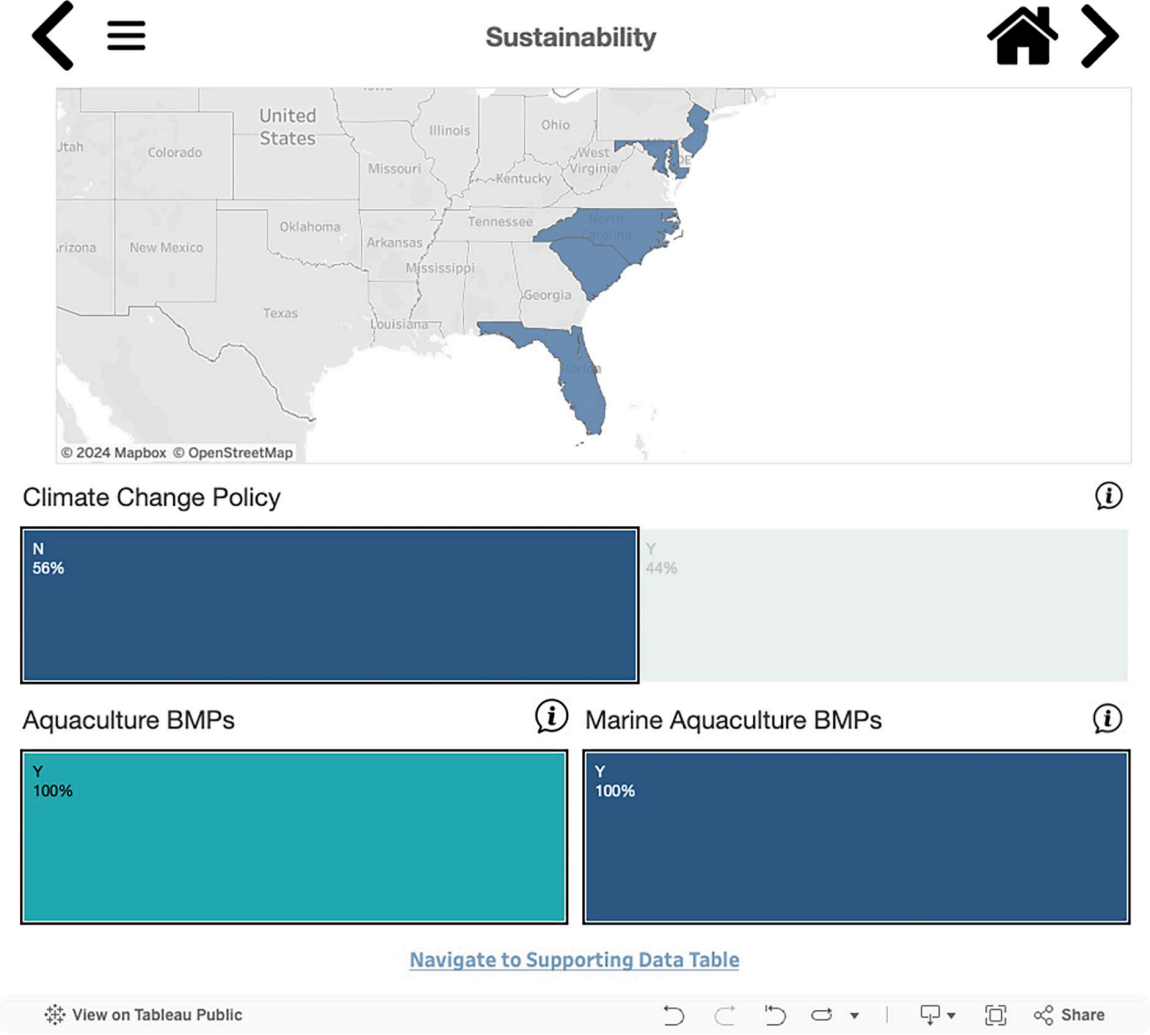
Example of visualization for specific policy groupings. The figure shows states that do not have climate change policies for their mariculture industries but do have aquaculture and mariculture Best Management Practices (BMPs). This Dashboard is made available under the Open Database License: http://opendatacommons.org/licenses/odbl/1.0/. Any rights in individual contents of the database are licensed under the Database Contents License: http://opendatacommons.org/licenses/dbcl/1.0/.

The Policy Specific Dashboards tool contains subsets of related data. We categorized the data based on nine key policy themes: legislation and regulations, management authority, biosecurity, capacity building, farming operations, leasing information, spatial management, sustainability, and tribal authority (see S1 Table). Each of these themes can be viewed in both chart and map form. These pages operate in a similar way to the Homepage, where attribute selections can be used to filter down the map views (Fig 5). Hovering over the information icon will display more information about that particular attribute. Hovering over the state within the map view will display summary information about the state. For more detail about the state policies, users can click on a state directly within the map views to navigate to the corresponding policy detail page for that state.

#### 2.4.4 Policy details links

**Fig 6:**
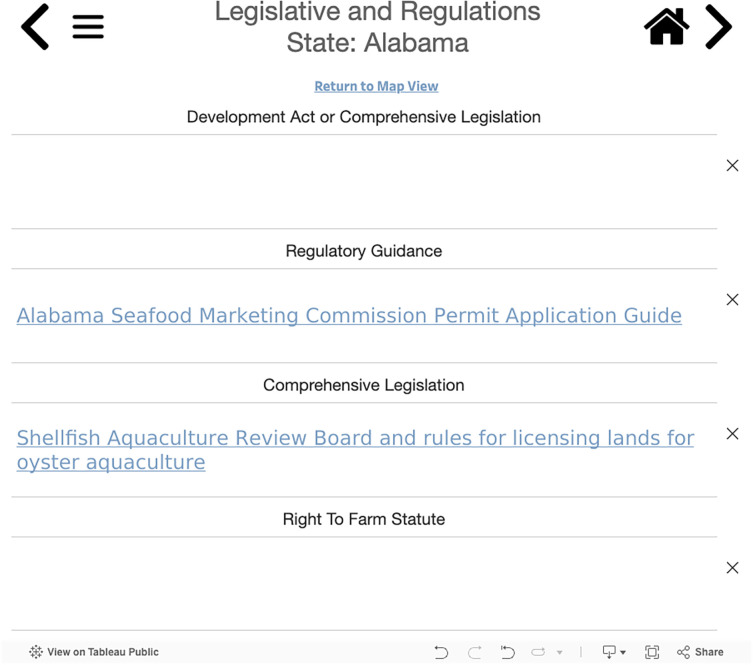
Example of visualization for state-specific policy details. The figure shows the underlying data for Alabama’s legislative and regulatory attributes.

The Policy Details Links tool allows users to navigate to the policy information for a specific state. Data for each state is categorized based on the same nine policy themes listed above. Users select a state from the drop-down menu and, once they have navigated to the relevant policy theme, they can view the underlying data for that state’s individual policy attributes (Fig 6). By hovering over the visible text, users will find full policy descriptions and sources for a given attribute. Clicking on the text will take users to the original source of the data.

## 3. Results

**Fig 7:**
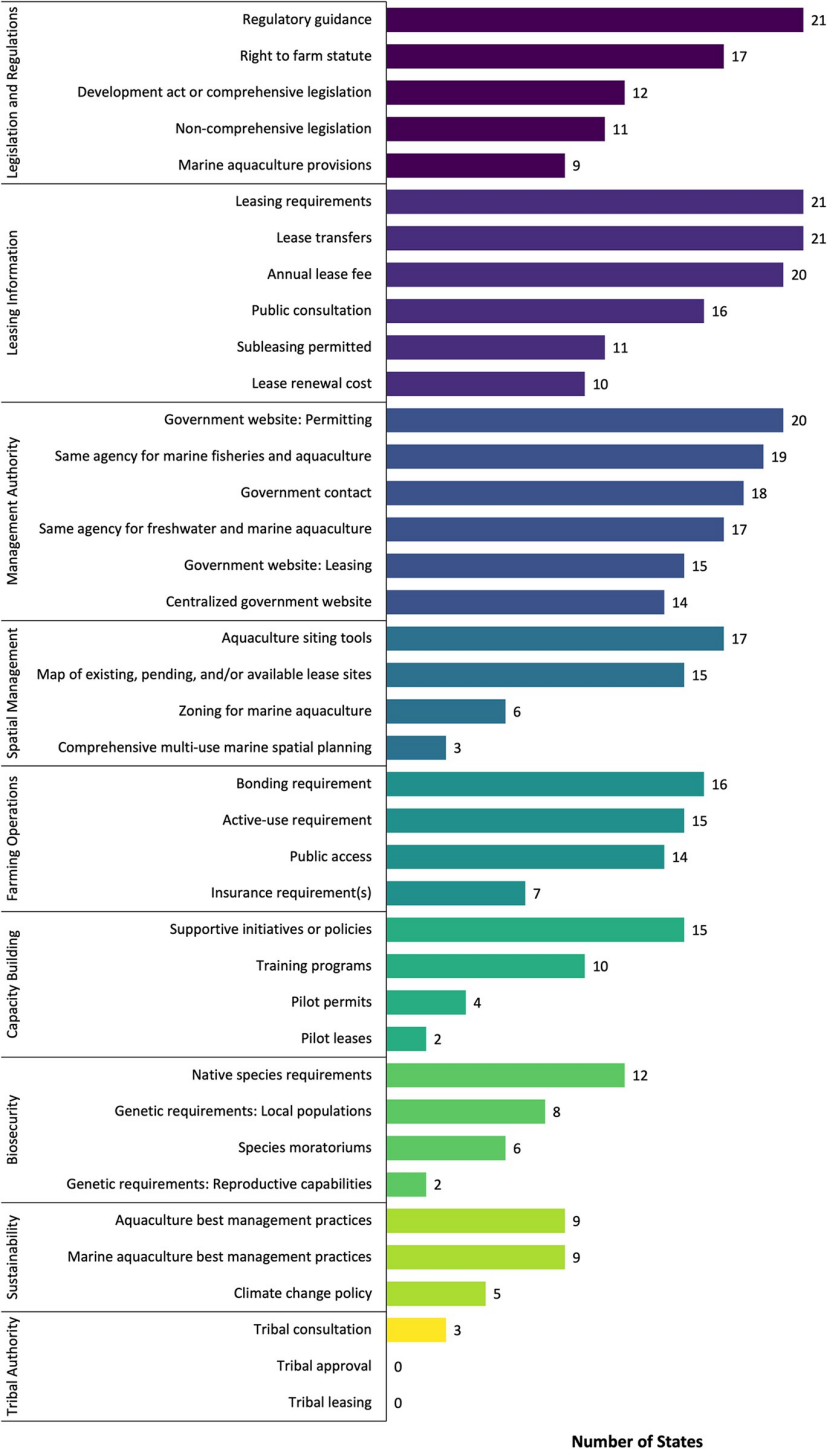
Frequency of mariculture policy attributes across the 23 coastal states. The attributes for ‘agency lead: permitting’, ‘agency lead: leasing’, and ‘maximum lease term’ are excluded as they do not consist of binary data.

The number of coastal states that have each policy attribute varied greatly (Fig 7). Policy attributes within the themes of legislation and regulation of mariculture activities, the leasing of ocean space for mariculture purposes, and management authority of the industry were most common across states. Individual states had between 26–69% of the policy attributes in the Dashboard. Regulatory guidance, leasing requirements, and the ability to transfer mariculture leases are the most represented policy attributes across states (N = 21). Meanwhile, policy attributes addressing biosecurity, sustainability, and tribal authority were least common. Only three states require mariculture permit applicants to consult with tribal governments and/or leadership regarding possible conflicts with traditional use., While no states require applicants to obtain formal approval from these tribal authorities, consulting with them can facilitate tribal support and avoid pushback during public hearings and/or comment periods.

## 4. Discussion

The Dashboard improves on previous efforts to synthesize state-level mariculture policy data in several ways. First, as a living database, the Dashboard will be updated frequently (at least once annually) through internal reviews and consultations with mariculture managers from each state, ensuring that the most up-to-date information is available. We designed the Dashboard using a public software platform—Tableau Public—that interfaces with Google sheets, so coding support is not needed, and we can update the Dashboard data easily and at limited financial cost. Second, the Dashboard is interactive and presents the policy data in multiple configurations, making it suitable to a broad range of user needs and objectives. Additionally, users will be able to download the dataset on which the Dashboard is built, encouraging broad exploration and analysis of mariculture policy. Lastly, the Dashboard was designed to allow for future expansion of the underlying dataset, which could entail the addition of new attributes (e.g., water quality standards, effluent regulations, riparian restrictions, farm subsidies), the modification of existing attributes, and the potential inclusion of U.S. territories or the Great Lakes states alongside the 23 coastal states. This flexibility means the Dashboard can evolve with the mariculture policy landscape.

Given the growing emphasis on mariculture as a means of diversifying coastal economies and expanding national seafood security [14–16], effective policy and regulatory mechanisms are critical to industry development [1, 2]. Further, an inefficient and uncoordinated policy landscape is costly and has resulted in the closure of farms and company operations in the U.S in favor of countries with more streamlined systems [6]. Compiling state-level mariculture policy data into a publicly-available online tool supports this goal by increasing legislative and regulatory transparency.

As states look to develop more comprehensive mariculture policy frameworks, the Dashboard can mobilize policy transfers, with states learning from what others have done and adapting policy approaches to their mariculture industry and their government structures [17]. Not having to start from scratch can expedite policy development and encourage the adaptation of innovative industry tools. For example, states interested in developing best management practices (BMPs) can identify other states with their own BMPs through the Dashboard, explore how these states have operationalized their BMP policy, and identify potential pathways for adopting and/or adapting these policy practices in their own states. Additionally, states seeking to encourage mariculture development can look up states with more mature industries and identify key policy mechanisms that can encourage and support industry growth.

From a research perspective, the Dashboard can be utilized to explore patterns in current mariculture policy across states, which can facilitate the identification of key policy gaps, enabling policies, or restrictive management frameworks that are impacting how the mariculture industry is developing and evolving. For example, utilizing the Dashboard, Ruff and Lester (2024) find that few states currently offer opportunities to trial, or ‘pilot’, mariculture activities on a small, short-term scale—an approach which has the potential to improve the economic, operational, and ecological feasibility of mariculture [18]. In addition to investigating patterns across states, researchers can delve into the specific policy landscape of a particular state, spurring questions about policy development processes, policy coherence, and regulatory frameworks. Our hope is that the Dashboard will facilitate diverse conversations and collaborations across policymakers, industry participants, regulators, and researchers and, in doing so, encourage new considerations for how to sustainably manage this increasingly vital industry.

## Supporting information

S1 Table State Marine Aquaculture Dashboard policy attributes and their definitions. Attributes are categorized based on 9 key policy ‘themes’.
(XLSX)
